# Central sensitivity to thyroid hormones is reduced in youths with overweight or obesity and impaired glucose tolerance

**DOI:** 10.3389/fendo.2023.1159407

**Published:** 2023-03-28

**Authors:** Procolo Di Bonito, Domenico Corica, Maria Rosaria Licenziati, Anna Di Sessa, Emanuele Miraglia del Giudice, Maria Felicia Faienza, Valeria Calcaterra, Francesca Franco, Giulio Maltoni, Giuliana Valerio, Malgorzata Wasniewska

**Affiliations:** ^1^ Department of Internal Medicine, “S. Maria delle Grazie” Hospital, Pozzuoli, Italy; ^2^ Department of Human Pathology of Adulthood and Childhood, University of Messina, Messina, Italy; ^3^ Neuro-endocrine Diseases and Obesity Unit, Department of Neurosciences, Santobono-Pausilipon Children’s Hospital, Napoli, Italy; ^4^ Department of Woman, Child and of General and Specialized Surgery, University of Campania “Luigi Vanvitelli”, Napoli, Italy; ^5^ Department of Precision and Regenerative Medicine and Ionian Area, University of Bari “Aldo Moro”, Bari, Italy; ^6^ Pediatric Department, Buzzi Children’s Hospital, Milano and Department of Internal Medicine, University of Pavia, Pavia, Italy; ^7^ Pediatric Department, Azienda Sanitaria Universitaria Friuli Centrale, Hospital of Udine, Udine, Italy; ^8^ Pediatric Unit, IRCCS Azienda Ospedaliero-Universitaria di Bologna, Bologna, Italy; ^9^ Department of Movement Sciences and Wellbeing, University of Napoli “Parthenope”, Napoli, Italy

**Keywords:** pediatric obesity, impaired glucose tolerance, prediabetes, thyroid hormones, sensitivity to thyroid hormones

## Abstract

**Background:**

Thyroid hormones (TH) play multiple effects on glucose metabolism. Some recent studies carried out in adult patients suggested an association between altered sensitivity to TH and type 2 diabetes, obesity, and metabolic syndrome. No studies are currently available on the presence of altered sensitivity to the action of TH in youths with prediabetes.

**Objective:**

To evaluate the relationship between sensitivity to TH and impaired glucose tolerance (IGT), impaired fasting glucose (IFG), or glycosylated hemoglobin (HbA1c) ≥ 5.7% in youths with overweight/obesity (OW/OB).

**Materials and methods:**

This cross-sectional study included 805 Caucasian youths with OW or OB (aged 6-18 years) recruited at seven Italian centers for the care of OW/OB. Individuals with TH out of the normal range of TH in each center were excluded. The fT3/fT4 ratio was evaluated to assess peripheral sensitivity, while TSH index (TSHI), Thyrotroph T4 Resistance Index (TT4RI), Thyroid Feedback Quantile-based Index (TFQI) and Parametric TFQI were calculated to assess central sensitivity.

**Results:**

Youths with IGT (n =72) showed higher levels of TSH (3.08 ± 0.98 vs 2.68 ± 0.98 mIU/L, P =0.001), TSHI (3.06 ± 0.51 vs 2.85 ± 0.53, P =0.001), TT4RI (46.00 ± 17.87 vs 38.65 ± 16.27, P <0.0001), TFQI [1.00 (0.97-1.00) vs 1.00 (0.99-1.00)], P=0.034), PTFQI (0.67 ± 0.20 vs 0.60 ± 0.22, P =0.007) compared to youths without IGT (n =733), independently of centers and age. No differences were observed for fT3/fT4-ratio. The others phenotypes of prediabetes were not associated with altered sensitivity to TH. Odds ratio of IGT raised of 1-7-fold for each increase of 1 mIU/L in TSH (P =0.010), 1 unit in TSH Index (P =0.004), TT4RI (P =0.003) or PTFQI (P =0.018), independently of centers, age, and prepubertal stage.

**Conclusion:**

IGT was associated with a reduced central sensitivity to TH in youths with OW/OB. Our finding suggests that IGT phenotype, known to be associated with an altered cardiometabolic risk profile, might also be associated with an impaired TH homeostasis in youths with OW/OB.

## 1 Introduction

Thyroid hormones (TH) play multiple effects on glucose metabolism, affecting insulin secretion and glucose uptake or output at different levels. TH stimulate insulin secretion by pancreatic β-cells and glucagon release by pancreatic α-cells and centrally modulate glucose production through a sympathetic pathway from the hypothalamic paraventricular nucleus to liver, independently of glucoregulatory hormones (1, 2). Furthermore, TH promote glucose absorption by enhancing gastrointestinal motility. In the liver they stimulate glucose output by increasing gluconeogenesis and glycogenolysis. Hepatic gluconeogenesis is also synergically stimulated by the lipolytic effect of TH in adipose tissue. Lastly, TH increase glucose uptake in skeletal muscle (1, 2).

Interestingly, both subclinical hypothyroidism and prediabetes/type 2 diabetes share a common metabolic abnormality represented by insulin resistance (IR) (3–5). In fact, it has been documented that TSH and free thyroxine (fT4) levels, still within normal range, correlate with IR and are associated with an increased risk of type 2 diabetes (3, 5). On the other hand, other studies highlighted that free triiodothyronine (fT3) and fT4, rather than TSH, are related to cardiovascular and metabolic risk factors in euthyroid subjects (4). These apparently contradictory results can be explained by the several effects that TH have on glucose metabolism (1, 2). Regarding the association between thyroid function and risk of prediabetes/type 2 diabetes, another aspect related both to central and peripheral sensitivity to the action of TH should be also considered. Some recent studies carried out in adult patients suggested a close association between altered sensitivity to TH and type 2 diabetes, obesity (OB) and metabolic syndrome (6, 7). A single pilot study carried out in prepubertal children with obesity documented that central and peripheral sensitivity to TH is significantly affected by the severity of overweight and the presence of IR (8). However, it has not yet been verified whether an altered sensitivity to TH is detectable in youths with prediabetes.

Aim of this cross-sectional study is to evaluate the association between sensitivity to TH and phenotypes of prediabetes in Caucasian children and adolescents with overweight (OW) or OB.

## 2 Materials and methods

### 2.1 Study population

**Figure 1:**
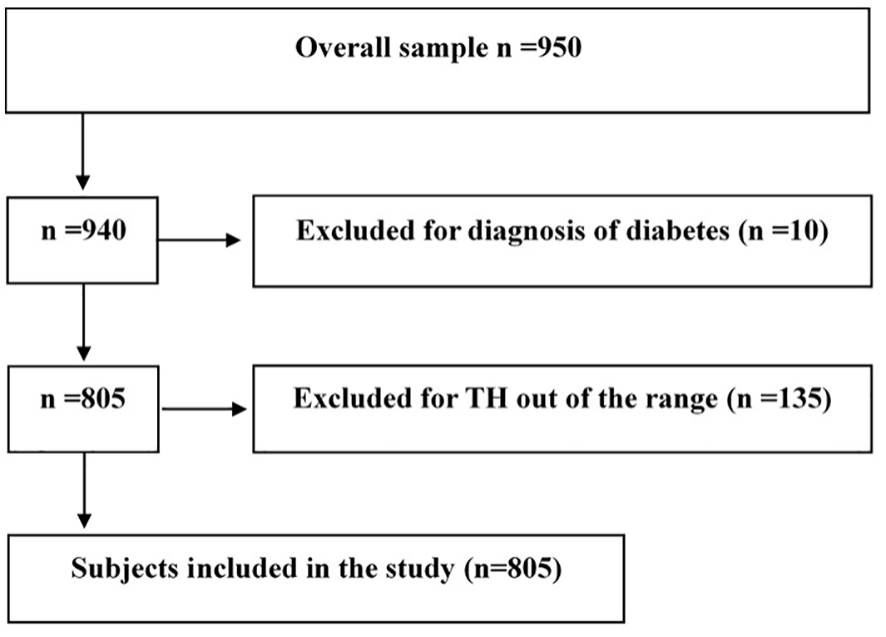
Flowchart of the selection of study participants. Thyroid hormones (TH).

This retrospective cross-sectional study was undertaken on behalf of the pediatric obesity study group of Italian Society for Pediatric Endocrinology and Diabetology (ISPED). Seven tertiary Italian centers for diagnosis and care of pediatric obesity provided records of children and adolescents with OW or OB (age 6-18 years) consecutively observed from June 2016 to June 2020, as previously described (9). Caucasian youths with OW or OB defined according to Italian BMI standards (the 75^th^ and 95^th^ percentiles, respectively) (10), TH within the normal range, thyroid peroxidase antibody (TPO-Ab) and thyroglobulin antibody (TG-Ab) negativity, data availability for fasting glucose and insulin, oral glucose tolerance test (OGTT), glycosylated hemoglobin (HbA1c), lipids, blood pressure (BP), were included in this study. Exclusion criteria were: thyroid diseases, subclinical hypothyroidism, genetic or endocrine obesity, chronic diseases, pharmacological treatment, type 2 diabetes, and type 1 diabetes. In the entire sample evaluated retrospectively, TH assay was available in 950 subjects. Ten youths with diagnosis of diabetes (fasting glucose >126 mg/dL or 2-hour glucose ≥200 mg/dL after OGTT or HbA1c ≥6.5%) were excluded (**Figure 1**). In addition, 135 subjects were excluded because they had TH values outside the normal range for each individual center. Finally, data of 805 youths were analyzed as reported in **Figure 1**. This study was approved by the Ethics Committee of the AORN Santobono-Pausilipon (reference number 22877/2020). The study was also in accordance with the 1975 Declaration of Helsinki, revised in 2013, and informed consent was obtained from the parents or tutors of all participants, as elsewhere described (8).

### 2.2 Measurements

Height and weight were measured in each center by a single expert operator; body mass index (BMI) was calculated as weight/height^2^, as previously described (9). BMI was transformed into standard deviation score (SDS), based upon the Italian BMI percentiles (10).

After a 12-hour fast, blood sampling was performed in each center’s centralized laboratory for TH, thyroid antibodies, fasting glucose (G_0_), insulin (IRI_0_), HbA1c, lipids. OGTT was performed as recommended by American diabetes association (ADA) using 1.75 g/kg of glucose up to a maximum of 75 g (11), and two-hour post-load glucose (G_120_) was analyzed. HbA1c was assessed by high performance liquid chromatography. Homeostatic model assessment index (HOMA-IR) was used to estimate IR by the following formula: (I_0_ [mU/L] x G_0_ [mmol/L])/22.5.

All laboratories belong to the Italian National Health System and are certified according to International Standards IS 000 (www.iso000.it/), undergoing to semi-annual quality controls and inter-lab comparisons.

### 2.3 Thyroid hormones and indices of sensitivity to thyroid hormones

The measurement of fT3, fT4 and TSH was performed by chemiluminescence immunoassay methods at each center. Each center’s laboratory normality reference ranges are reported in the supplementary materials (**Table S1**). The records of patients included in this study ranged according to the lower and upper limits of the method adopted for TH assessment at each center. To ensure the exclusion of individuals with subclinical hypothyroidism, youths with TSH values >4.9 mIU/L were excluded (12, 13). Indices expressing peripheral and central sensitivity to TH were calculated. In particular, the fT3/fT4 ratio was evaluated to assess peripheral sensitivity, while TSH index (TSHI), Thyrotroph T4 Resistance Index (TT4RI), Thyroid Feedback Quantile-based Index (TFQI), Parametric Thyroid Feedback Quantile-based Index (PTFQI) were calculated to assess central sensitivity, as previously described (6).

TSHI was calculated as ln TSH (mIU/L) + 0.1345 x fT4 (pmol/L); TT4RI was calculated as fT4 (pmol/L) x TSH (mIU/L); TFQI was assessed as cdf fT4 - (1 - cdf TSH), where cdf is for empirical cumulative distribution function (cdf) to hormone concentration; PTFQI was assessed according to the following formula: Φ((FT4 – μ_FT4_/σ _FT4_) – (1 – Φ (ln TSH - μ _ln TSH_/σ _ln TSH_)), where μ_FT4_ = 10.075, σ _FT4_ = 2.155, μ _ln TSH_ = 0.4654, σ _ln TSH_ = 0.7744 (6).

An increase in TSHI, TT4RI, TFQI, and PTFQI indices corresponds to a decrease in central sensitivity to TH. On the other hand, an increase in fT3/fT4-ratio correspond to an increase in peripheral sensitivity to TH.

### 2.4 Definitions

Prepubertal stage was defined by Tanner stage I (no breast development in girls and testicular volume below 4 ml in boys). OW and OB were defined on the basis of the Italian BMI standards (the 75^th^ and 95^th^ percentiles, respectively) (10). American Diabetes Association diagnostic criteria were adopted to classify participants with phenotypes of prediabetes (11). Impaired fasting glucose (IFG) was defined by fasting blood glucose ≥100 mg/dL but <126 mg/dL; impaired glucose tolerance (IGT) was defined by 2-h blood glucose ≥140 mg/dL but <200 mg/dL. High HbA1c was defined by HbA1c ≥5.7% (≥ 39 mmol/mol) but <6.5% (<48 mmol/mol). Prediabetes was defined as any of the following phenotypes: IGT or IFG or high HbA1c (11). Family history of type 2 diabetes was defined by the presence of type 2 diabetes in at least one among the first- or second-degree relatives.

### 2.5 Statistical analyses

Data were expressed as mean ± standard deviation (SD), numbers, proportions (%) and 95% confidence interval (CI). Variables with skewed distribution (i.e., HOMA-IR, TG/HDL-ratio and TFQI) were log transformed for the analysis and expressed in the tables as median and interquartile range. Mean values and proportions were compared after correction for centers. All the values of the TH and their derived indices shown in the tables were analyzed by ANCOVA adjusted for centers and age. Distribution of categorical variables was compared by χ^2^. The association between IGT and indices of sensitivity to TH was tested using logistic regression analysis adjusted for centers, age, and prepubertal stage (Model 1) and centers, age, prepubertal stage, family history of type 2 diabetes, HOMA-IR, TG/HDL ratio, systolic BP, obesity, IFG and high HbA1c (Model 2). A two-sided *P* value less than 0.05 was considered statistically significant. The statistical analysis was performed with IBM SPSS Statistics, version 20.0 (IBM Corp., Armonk, New York, USA).

## 3 Results

Clinical and biochemical characteristics of the study population are shown in **Table 1**. A higher prevalence of prepubertal stage, higher systolic BP and fasting glucose was observed in males compared to females, while higher HOMA-IR values were observed in females. No sex differences were observed in the prevalence of obesity, IFG, IGT or high HbA1c. With regard to TH, higher fT3 levels and fT3/fT4 ratio were documented in males compared to females, independently of centers and age. No sex differences were observed with regard to TSH values ​​and derived indices of central sensitivity to TH.

**Table 1:** Description of the sample as a whole and by sex.

*n*	All	Boys	Girls	*P* value
	*805*	*404*	*401*	
Age, years	11.3 ± 2.5	11.3 ± 2.5	11.3 ± 2.6	0.807*
Prepubertal stage, n (%)	120 (14.9)	79 (19.6)	41 (10.2)	<0.0001*
BMI (kg/m^2^)	30.7 ± 5.4	30.8 ± 5.1	30.5 ± 5.6	0.349*
BMI-z score (SDS)	2.3 ± 0.6	2.3 ± 0.6	2.3 ± 0.6	0.950*
Obesity, n (%)	714 (88.7)	367 (90.8)	347 (86.5)	0.094*
Family history of T2D, (%)	455 (56.5)	230 (56.9)	225 (56.1)	0.840*
G_0_ (mg/dL)	86.2 ± 10.1	87.1 ± 10.1	85.3 ± 10.1	0.009*
G_120_ (mg/dL)	110.2 ± 20.6	110.8 ± 19.5	109.7 ± 21.7	0.531*
HbA1c, (%)	5.3 ± 0.4	5.3 ± 0.4	5.3 ± 0.4	0.834*
HbA1c (mmol/mol)	34.3 ± 4.0	34.3 ± 4.0	34.3 ± 4.1	0.834*
HOMA-IR	3.5 (2.4-5.0)	3.3 (2.3-4.8)	3.8 (2.4-5.5)	0.031*
TG/HDL ratio	1.79 (1.28-2.65)	1.78 (1.25-2.61)	1.84 (1.30-2.67)	0.480*
Systolic BP (mmHg)	112.3 ± 13.9	113.6 ± 14.0	110.9 ± 13.7	0.009*
Diastolic BP (mmHg)	66.4 ± 9.5	66.6 ± 9.1	66.3 ± 10.0	0.732*
fT3 (pmol/L)	6.2 ± 1.0	6.3 ± 0.9	6.0 ± 1.0	<0.0001**
fT4 (pmol/L)	14.4 ± 2.5	14.4 ± 2.5	14.3 ± 2.5	0.451**
fT3/fT4 ratio	0.44 ± 0.10	0.45 ± 0.09	0.43 ± 0.10	0.020**
TSH (mIU/L)	2.74 ± 0.98	2.69 ± 1.00	2.69 ± 1.00	0.445**
TSH Index	2.86 ± 0.53	2.89 ± 0.53	2.84 ± 0.54	0.189**
TT4RI	39.3 ± 15.5	40.0 ± 17.0	38.6 ± 16.0	0.235**
TFQI	0.99 (0.97-1.00)	0.99 (0.98-1.00)	0.99 (0.97-1.00)	0.191**
PTFQI	0.61 ± 0.22	0.61 ± 0.22	0.60 ± 0.22	0.353**
IGT, n (%)	72 (8.9)	32 (7.9)	40 (10)	0.283*
IFG, n (%)	73 (9.1)	43 (10.6)	30 (7.5)	0.160*
HbA1c ≥5.7%	133 (16.5)	67 (16.6)	66 (16.5)	0.910*

Data are expressed as mean ± standard deviation, median (IQ range), n (%). *P value adjusted for age. **P value adjusted for centers and age.

BMI, body mass index; SDS, standard deviation score; G_0_, fasting glucose; G_120_, glucose at 120’ during oral glucose tolerance test; HOMA-IR , homeostasis model assessment, TG/HDL ratio, triglycerides to HDL ratio; BP, blood pressure; fT4, free thyroxine; fT3, free triiodothyronine; TSH, thyroid stimulating hormone; TT4RI, Thyrotroph T4 Resistance Index; TFQI, Thyroid Feedback Quantile-based Index; PTFQI Parametric Thyroid Feedback Quantile-based Index; IGT, impaired glucose tolerance.

Youths with IGT were older, showed a lower prevalence of prepubertal stage and a higher systolic BP, HOMA-IR, TG/HDL ratio, independently of centers. Furthermore, TSH, TSH Index, TT4RI, TFQI and PTFQI values were significantly higher in subjects with IGT than in youth without IGT, independently of centers and age (**Table 2**). No differences were observed for fT3/fT4-ratio between IGT and non-IGT. A similar result was observed in youths with isolated IGT (n =42) as compared to normoglycemic youths without IFG and high HbA1c (n =583) as reported in the Supplementary Materials (**Table S2**).

**Table 2:** Anthropometric, clinical, biochemical and thyroid variables by IGT.

n =805	No IGT (n =733)	IGT (n =72)	P value
Male sex, n (%)	372 (50.8)	32 (44.4)	0.282*
Prepubertal stage, n (%)	115 (15.7)	5 (6.9)	0.053*
Obesity, n (%)	647 (88.3)	67 (93.1)	0.191*
Family history, n (%)	405 (55.3)	50 (69.4)	0.011*
Age (years)	11.3 ± 2.5	11.9 ± 2.4	0.037*
BMI, Kg/m^2^	30.6 ± 5.3	31.6 ± 5.8	0.137*
BMI-SDS	2.3 ± 0.6	2.4 ± 0.6	0.198*
HOMA-IR	3.4 (2.3-4.9)	4.8 (4.6-7.8)	<0.0001*
TG/HDL ratio	1.8 (1.2-2.6)	2.0 (1.6-3.2)	0.001*
Systolic BP (mmHg)	111.9 ± 13.9	115.6 ± 14.0	0.044*
Diastolic BP (mmHg)	66.1 ± 9.5	67.3 ± 9.8	0.455*
fT3 (pmol/L)	6.17 ± 0.97	6.30 ± 0.84	0.132**
fT4 (pmol/L)	14.36 ± 2.49	14.72 ± 2.65	0.156**
fT3/fT4 ratio	0.44 ± 0.10	0.44 ± 0.10	0.898**
TSH (mIU/L)	2.68 ± 0.98	3.08 ± 0.98	0.001**
TSH Index	2.85 ± 0.53	3.06 ± 0.51	0.001**
TT4RI	38.65 ± 16.27	46.00 ± 17.87	<0.0001**
TFQI	1.00 (0.97-1.00)	1.00 (0.99-1.00)	0.027**
PTFQI	0.60 ± 0.22	0.67 ± 0.20	0.004**

Data are expressed as mean ± standard deviation, median (IQ range), n (%).

*P value adjusted for centers, **P value adjusted for centers and age.

BMI, body mass index; SDS, standard deviation score; G_0_, fasting glucose; G_120_, glucose at 120’ during oral glucose tolerance test; HOMA-IR , homeostasis model assessment, TG/HDL ratio, triglycerides to HDL ratio; BP, blood pressure; fT4, free thyroxine; fT3, free triiodothyronine; TSH, thyroid stimulating hormone; TT4RI, Thyrotroph T4 Resistance Index; TFQI, Thyroid Feedback Quantile-based Index; PTFQI Parametric Thyroid Feedback Quantile-based Index; IGT, impaired glucose tolerance.

Youths with IFG showed higher levels of HOMA-IR, TG/HDL-ratio and diastolic BP with respect to youths without IFG (**Table 3**). No differences in any parameter were found between youths with HbA1c ≥5.7% and HbA1c <5.7% (**Table 3**). Neither IFG nor HbA1c ≥5.7% phenotypes showed differences in TSH levels or markers of sensitivity to TH (**Table 3**). The Odds of IGT increased f 1-7-fold for each increase of 1 mIU/L in TSH (*P*=0.008) or 1 unit in TSH Index (*P*=0.003), TT4RI (*P*=0.002) or PTFQI (*P* =0.016), independently of centers, age and prepubertal stage (**Table 4**). This result was confirmed in the Model 2 of logistic regression analysis, which was also adjusted for the following variables: family history of type 2 diabetes, HOMA-IR, TG/HDL ratio, systolic BP, obesity, IFG and high HbA1c (**Table 4**).

**Table 3:** Anthropometric, clinical, biochemical and thyroid variables by IFG and A1c ≥5.7%.

*n =805*	No IFG (n =732)	IFG (n =73)	*P* value	HbA1c <5.7% (n =672)	HbA1c ≥5.7% (n =133)	*P* value
Male sex, n (%)	361 (49.3)	43 (58.9)	0.158*	337 (50.1)	67 (50.4)	0.910*
Prepubertal stage, n (%)	111 (15.2)	9 (12.3)	0.505*	99 (14.7)	21 (15.8)	0.628*
Family history, n (%)	411 (56.1)	44 (60.3)	0.216*	397 (59.1)	58 (43.6)	0.536*
Obesity, n (%)	648 (88.5)	66 (90.4)	0.438*	591 (87.9)	123 (92.5)	0.154*
Age (years)	11.3 ± 2.5	11.6 ± 2.6	0.498*	11.4 ± 2.5	11.1 ± 2.6	0.100*
BMI, Kg/m^2^	30.6 ± 5.4	30.9 ± 5.5	0.689*	30.7 ± 5.3	30.5 ± 5.7	0.743*
BMI-SDS	2.3 ± 0.6	2.3 ± 0.6	0.847*	2.3 ± 0.6	2.4 ± 0.6	0.866*
HOMA-IR	3.4 (2.3-4.9)	4.5 (3.1-6.9)	<0.0001*	3.4 (2.3-4.9)	4.1 (2.6-6.2)	0.086*
TG/HDL ratio	1.8 (1.3-2.6)	2.0 (1.5-3.1)	0.035*	1.8 (1.3-2.7)	1.8 (1.3-2.6)	0.404*
Systolic BP (mmHg)	111.9 ± 14.0	115.8 ± 13.0	0.058*	112.2 ± 13.8	112.6 ± 14.90	0.501*
Diastolic BP (mmHg)	66.2 ± 9.5	69.3 ± 9.8	0.015*	66.5 ± 9.4	66.1 ± 10.2	0.302*
fT3 (pmol/L)	6.16 ± 0.96	6.34 ± 0.89	0.134**	6.15 ± 0.96	6.31 ± 0.92	0.260**
fT4 (pmol/L)	14.35 ± 2.46	14.87 ± 2.84	0.045**	14.42 ± 2.51	14.30 ± 2.48	0.999**
fT3/fT4 ratio	0.44 ± 0.09	0.44 ± 0.11	0.991**	0.44 ± 0.09	0.45 ± 0.10	0.313**
TSH (mIU/L)	2.71 ± 0.99	2.73 ± 0.99	0.804**	2.70 ± 1.00	2.79 ± 0.96	0.283**
TSH Index	2.86 ± 0.53	2.94 ± 0.61	0.146**	2.86 ± 0.54	2.89 ± 0.50	0.308**
TT4RI	39.09 ± 16.25	41.54 ± 19.17	0.153**	39.09 ± 16.52	40.43 ± 16.66	0.243**
TFQI	1.00 (0.97-1.00)	1.00 (0.97-1.00)	0.756**	1.00 (0.97-1.00)	1.00 (0.98-1.00)	0.330**
PTFQI	0.61 ± 0.22	0.61 ± 0.24	0.715**	0.61 ± 0.22	0.62 ± 0.20	0.363**

Data are expressed as mean ± standard deviation, median (IQ range), n (%).

*P value adjusted for centers. **P value adjusted for centers and age.

BMI, body mass index; SDS, standard deviation score; G_0_, fasting glucose; G_120_, glucose at 120’ during oral glucose tolerance test; HOMA-IR , homeostasis model assessment, TG/HDL ratio, triglycerides to HDL ratio; BP, blood pressure; fT4, free thyroxine; fT3, free triiodothyronine; TSH, thyroid stimulating hormone; TT4RI, Thyrotroph T4 Resistance Index; TFQI, Thyroid Feedback Quantile-based Index; PTFQI Parametric Thyroid Feedback Quantile-based Index; IGT, impaired glucose tolerance.

**Table 4:** ORs (95% CI) of IGT for 1 mIU/L increase of TSH or 1 unit of TSHI, TT4RI or PTFQI.

	No IGT	IGT	*P* value
TSH (increase of 1 mIU/L)			
Model 1	1.00	1.52 (1.18-1.96)	0.001
Model 2	1.00	1.41 (1.08-1.84)	0.010
TSH Index (increase of 1 unit)			
Model 1	1.00	2.44 (1.45-4.09)	0.001
Model 2	1.00	2.15 (1.26-3.68)	0.005
TT4RI (increase of 1 unit)			
Model 1	1.00	1.03 (1.01-1.04)	<0.0001
Model 2	1.00	1.02 (1.01-1.04)	0.004
PTFQI (increase of 1 unit)			
Model 1	1.00	7.49 (1.95-28.78)	0.003
Model 2	1.00	4.92 (1.25-19.39)	0.023

Model 1: P value adjusted for centers, age and prepubertal stage.

Model 2: P value adjusted for centers, age, prepubertal stage, family history of type 2 diabetes,

HOMA-IR, TG/HDL ratio, systolic BP, obesity, IFG and high HbA1c.

TSH, thyroid stimulating hormone; TT4RI, Thyrotroph T4 Resistance Index; TFQI, Thyroid Feedback Quantile-based Index; PTFQI Parametric Thyroid Feedback Quantile-based Index; IGT, impaired glucose tolerance.

## 4 Discussion

This study provides evidence that among the three phenotypes of prediabetes, only IGT is associated with reduced sensitivity to the central action of TH in euthyroid youths with OW/OB. To the best of our knowledge, this is the first study to investigate the relationship between sensitivity to TH and prediabetes in children and adolescents with OW/OB confirming the results of the few studies performed on adults (6, 7). Laclaustra et al., to clarify the role of TH sensitivity in impairment of glucose metabolism, demonstrated that an increased central resistance to TH was associated with OB, type 2 diabetes, and the risk of diabetes-related deaths in adults (6). These authors suggested that TH resistance might be an expression of energy balance impairment underlying type 2 diabetes (6). These results were partially confirmed by Mehran et al. in an Iranian population (7). Consistently, Nie et al. in a cross-sectional study suggested that decreased sensitivity to TH was positively related to the level of the adipocyte fatty acid-binding protein (A-FABP), a fat-derived protein that has been positively correlated to glucose dysregulation, metabolic syndrome, and vascular disease (14). Conversely, Liu et al. demonstrated that decreased central sensitivity to TH is associated with lower risk of prediabetes defined as IFG or increased HbA1c (from 5.7% to 6.4%) (15).

The only pediatric evaluation of the relationship between sensitivity to TH and glucose metabolism was carried out in a pilot study of an Italian selected population of prepubertal, euthyroid, children with obesity, which did not include any young people recruited in the present study (8). These Authors reported a significant association between insulin sensitivity and either central or peripheral TH resistance (8). Results of the present study, that include euthyroid youths at various stages of pubertal development, extend our knowledges about central TH sensitivity in youths with different prediabetes phenotypes. In fact, the main aim of the present study is to evaluate the association between TH sensitivity and the different phenotypes of prediabetes, defined by IFG, IGT or high HbA1c, in children with OW/OB. We demonstrate that a reduced central sensitivity to TH was associated only to IGT in these subjects. It has previously been documented that the IGT phenotype is characterized by a worse cardio-metabolic risk profile compared to IFG (16) or high HbA1c (9) in youths with OW/OB. In the present study we demonstrated that abnormalities in TH sensitivity are independently of cardio-metabolic risk. Therefore, it is possible that this association may be due to other unexplored factors.

We speculated that the association between impaired glucose metabolism and altered sensitivity to TH, previously reported in adults, may involve children as well. Altered sensitivity to the action of TH could be an early consequence of allostatic overload in the obese individual (17), even before overt or subclinical alteration of the TH profile occurs. Both in adults and children with OB, TH pattern is frequently characterized by normal-high/high TSH levels, normal-low/normal FT4 and normal-high/high FT3 levels (18). These biochemical findings are an example of allostatic adaptations of the hypothalamic-pituitary-thyroid hormonal axis, i.e. a dynamic reaction of the body to maintain stability through change (called allostasis) (5). In the presence of excessive weight, the biological system’s functioning adapts to a state where energy intake and storage exceeds the body’s energy demand (allostatic overload). This allostatic adaptation thus maintains stability in a condition of energy overload typical of OB, but also promotes changes that may not be favorable in the long term. Indeed, the increase in TSH may contribute to the well-known inflammatory state associated with OB by promoting the synthesis of pro-inflammatory cytokines by adipose tissue (19–21). Furthermore, TSH levels, in synergy with leptin, indirectly promote IR through the synthesis of pro-inflammatory cytokines in adipocytes (22).

Therefore, it is plausible that allostatic-adaptive thyroid responses to obesity may already influence metabolic outcome not only in the presence of overt or subclinical hypothyroidism, but also in the presence of a euthyroid state, affecting the risk of early cardio-metabolic alterations, including IGT (5, 23).

Due to the cross-sectional design of our study, the directionality of the association between IGT and altered sensitivity to TH cannot be fully explained, as it occurs in studies carried out in adults with type 2 diabetes.

The cross-sectional, retrospective, design represents a limitation of our study. In addition, examining only young Caucasians with OW/OB limits the extendibility of the results to the general population. On the other hand, our study has significant strengths represented by a relatively large and well characterized sample of euthyroid Caucasian children and adolescents.

In conclusion, our study documented the presence of an association between an altered sensitivity to TH and IGT in children and adolescents with OW/OB, without any impairment of fT3 and fT4. The results of this preliminary study suggest that the pathogenesis of IGT could also be influenced by central resistance to TH in youths with obesity, although the opposite cannot be excluded. Since prediabetic phenotypes can change over time, our results should be interpreted with caution and confirmed in prospective studies. The results of this study suggest new directions for future research to understand the pathophysiological link between prediabetes and TH sensitivity.

## Data availability statement

The raw data supporting the conclusions of this article will be made available by the authors, without undue reservation.

## Ethics statement

The studies involving human participants were reviewed and approved by Ethics Committee of the AORN Santobono-Pausilipon, Naples, Italy. Written informed consent to participate in this study was provided by the participants’ legal guardian/next of kin.

## Author contributions

Conceptualization, PB and DC; methodology, PB, DC, MW and GV; software, PB; formal analysis, PB; investigation, DC, ML, AS, EM, MF, VC, FF, GM and MW; resources, DC, ML, AS, EM, MF, VC, FF, GM and MW; data curation, PB, DC, GV and MW; writing—original draft preparation, PB, DC, GV and MW. All authors contributed to the article and approved the submitted version. 
