# Supplementary material for: Central sensitivity to thyroid hormones is reduced in youths with overweight or obesity and impaired glucose tolerance
Source: Front Endocrinol (Lausanne). 2023 Mar 28;14:1159407. doi: 10.3389/fendo.2023.1159407 (PMC10092349; doi:10.3389/fendo.2023.1159407)
Supplement: Supplementary file 1 [file DataSheet_1.pdf]

Supplementary Material

Table S1. Normal range of thyroid hormones by centers

Center	City (Age)	fT3 pmol/L	fT4 pmol/L	TSH (mIU/L)
Center 1	Messina	3.1-6.8	12.0–22.0	0.3-5.0
Center 2	Santobono	3.8-8.0	11.8-21.9	0.5-4.3
Center 3	Napoli “Vanvitelli” (5-7 years)	1.5-10.3	5.7-29.5	0.10-5.9
Center 3	Napoli “Vanvitelli” (8-16 years)	1.7-8.1	8.9-21.8	0.2-6.1
Center 3	Napoli “Vanvitelli” (>16 years)	2.3-9.1	6.7-20.3	0.2-6.1
Center 4	Bari	3.5-6.5	9.8-18.8	0.4-3.74
Center 5	Pavia	3.5-6.3	9.0-19.3	0.5-4.2
Center 6	Udine (5-12 years)	5.1-7.4	11.1-18.1	0.7-4.2
Center 6	Udine (≥13 years)	4.7-7.2	10.7-18.4	0.5-4.2
Center 7	Bologna	3.7-6.1	7.1-15.4	0.25-4.5

Table S2. Comparison between young people with normoglycemia (i.e., without IFG or high HbA1c) and isolated IGT

	**Normoglycemia**	**Isolated IGT**	***P* value***
**n =625**	**583**	**42**	
fT3 (pmol/L)	6.14±0.98	6.31±0.96	0.224
fT4 (pmol/L)	14.37±2.45	14.53±2.72	0.693
fT3/fT4 ratio	0.44±0.09	0.45±0.10	0.208
TSH (mIU/L)	2.68±0.99	3.07±1.08	0.002
TSH Index	2.84±0.54	3.01±0.56	0.029
TT4RI	38.58±16.13	45.26±19.02	0.003
TFQI	1.00 (0.97-1.00)	1.00 (0.99-1.00)	0.079
PTFQI	0.60±0.22	0.66±0.22	0.050

Data are expressed as mean±standard deviation, median (IQ range).
**P* value adjusted for centers and age
